# Repurposing of approved drugs for targeting CDK4/6 and aromatase protein using molecular docking and molecular dynamics studies

**DOI:** 10.1371/journal.pone.0291256

**Published:** 2023-09-08

**Authors:** Fatima A. yousif, Abdulrahim A. Alzain, Alhafez M. Alraih, Walaa Ibraheem

**Affiliations:** 1 Department of Pharmaceutical Chemistry, Faculty of Pharmacy, University of Gezira, Wad Madani, Sudan; 2 Department of Chemistry, College of Science and Arts, Mohail Aseer, King Khalid University, Abha, Kingdom of Saudi Arabia; Ahram Canadian University, EGYPT

## Abstract

Breast cancer is a leading cause of cancer-related morbidity and mortality worldwide, with the highest incidence among women. Among the various subtypes of breast cancer, estrogen-receptor positive (ER+) is the most diagnosed. Estrogen upregulates cyclin D1, which in turn promotes the activity of CDK4/6 and facilitates cell cycle progression. To address this, the first-line treatment for ER+ breast cancer focuses on inhibiting estrogen production by targeting aromatase, the enzyme responsible for the rate-limiting step in estrogen synthesis. Thus, combining CDK4/6 inhibitors with aromatase inhibitors has emerged as a crucial treatment strategy for this type of breast cancer. This approach effectively suppresses estrogen biosynthesis and controls uncontrolled cell proliferation, significantly improving overall survival rates and delayed disease progression. This study aimed to identify compounds that are likely to inhibit CDK4/6 and aromatase simultaneously by using a structure-based drug design strategy. 12,432 approved and investigational drugs were prepared and docked into the active site of CDK6 using HTVS and XP docking modes of Glide resulting in 277 compounds with docking scores ≤ -7 kcal/mol. These compounds were docked into aromatase enzyme using XP mode to give seven drugs with docking scores≤ -6.001 kcal/mol. Furthermore, the shortlisted drugs were docked against CDK4 showing docking scores ranging from -3.254 to -8.254 kcal/mol. Moreover, MM-GBSA for the top seven drugs was calculated. Four drugs, namely ellagic acid, carazolol, dantron, and apomorphine, demonstrated good binding affinity to all three protein targets CDK4/6 and aromatase. Specifically, they exhibited favourable binding free energy with CDK6, with values of -51.92, -53.90, -50.22, and -60.97 kcal/mol, respectively. Among these drugs, apomorphine displayed the most favourable binding free energy with all three protein targets. To further evaluate the stability of the interaction, apomorphine was subjected to a 100 ns molecular dynamics simulation with CDK6. The results indicated the formation of a stable ligand-protein complex. While the results obtained from the MM-GBSA calculation of the binding free energies of the MD conformations of apomorphine showed less favourable binding free energy compared to that obtained post-docking. All these computational findings will provide better structural insight for the development of CDK4/6 and aromatase multi-target inhibitors.

## 1. Introduction

Breast cancer is the most commonly diagnosed cancer among women worldwide [1], with the estrogen receptor-positive (ER+) subtype being the most prevalent [2]. Estrogen plays a critical role in the development of breast cancer in both pre-and postmenopausal women [3]. Consequently, targeting the estrogen signalling pathway has proven to be a successful strategy in treating this type of breast cancer [4]. The initial drug used to counter this pathway is tamoxifen, a selective estrogen receptor modulator (SERM). However, tamoxifen has undesirable side effects and provides incomplete blockade of estrogen, leading to the development of aromatase enzyme inhibitors (AIs) [5]. These inhibitors are effective and well-tolerated compared to tamoxifen [3]. AIs are categorized as steroidal and non-steroidal, with exemestane, anastrozole, and letrozole being the most commonly used AIs to treat estrogen receptor-positive breast cancer in postmenopausal women [6, 7].

Uncontrolled cell proliferation and the development of cancer are consequences of accelerated cell cycle progression. The cell cycle consists of four sequential phases: G1 (pre-DNA synthesis), S (DNA synthesis), G2 (pre-division), and M (cell division). The transition through these phases is regulated by different series of kinase activities [8]. Cyclin-dependent kinases (CDKs), a family of serine-threonine kinases, along with their protein partners called cyclins, are responsible for the regulation of the cell cycle. CDK4 and CDK6 are initiators of the transition from the G1 phase to the S phase [9]. Dysregulation of the cyclin D1-CDK4/6-Rb signalling cascade has been observed in breast cancer and other malignancies and is associated with poor prognosis and increased metastasis. Estrogen signalling induces cyclin D1, which enhances CDK4/6 activity and contributes to cancer progression [10]. The multi-targeting of CDK4/6 and aromatase inhibit estrogen biosynthesis and uncontrolled cell proliferation. Thus, has become the cornerstone of treatment for hormone receptor (HR)-positive, human epidermal growth factor receptor-2 (HER2)-negative metastatic breast cancer [11]. This combined approach has significantly delayed disease progression and improved overall survival rates [12].

Identification of new targets for older drugs is less likely to fail in future clinical trials due to their established clinical safety and known toxic properties, which are the primary causes of drug failures [13]. Drug repurposing refers to the process of discovering new therapeutic indications for existing drugs to enhance their productivity and maximize their potential utilization [14]. *In silico* drug, repurposing has gained global attention, leveraging the availability of bioinformatics and computational resources, resulting in time and cost savings [15].

The combination of CDK4/6 and aromatase inhibitors is the first-line treatment for HR-positive, HER2-negative metastatic breast cancer. To the best of our knowledge, there are no such multi-targeted inhibitors for targeting these enzymes. Thus, the objective of this study is to identify agents that potentially inhibit CDK4/6 and aromatase by employing the structure-based design strategy. Multi-step molecular docking, molecular mechanics energies combined with generalized born and surface area (MM-GBSA), and molecular dynamics techniques.

## 2. Materials and methods

All computational studies were performed using the Maestro version 12.8 of the Schrödinger suite (https://www.schrodinger.com). Molecular dynamics simulations were carried out using Desmond version 6.5, developed by D.E. Shaw Research [16].

### 2.1 Protein preparation

The crystallographic structures of CDK4 (PDB ID: 2W96), CDK6 (PDB ID: 5L2S), and aromatase enzyme (PDB ID: 3S79) were retrieved from the Protein Data Bank (PDB) database maintained by RCSB (http://www.rcsb.org). To prepare the proteins, the protein preparation wizard module from the Schrödinger software package was utilized. The following steps were performed on each protein: hydrogen atoms were added, and water molecules were removed, except in the case of CDK6 where they were retained due to their importance in inhibitor interactions [17]. Additionally, hydrogen bonds were introduced, and missing side chains and loops were filled. Subsequently, the pre-processed structures underwent optimization and minimization using the OPLS4 force field [18].

### 2.2 Grid generation

For CDK6 (PDB ID: 5L2S) and aromatase (PDB ID: 3S79), the binding cavities were defined using the receptor grid generation module around the bound ligands. In CDK6, the binding cavity was defined around the co-crystallized ligand abemaciclib, while in aromatase, it was defined around the co-crystallized ligand androstenedione. However, in CDK4 (PDB ID: 2W96), there was no co-crystallized inhibitor available. Therefore, the range of ATP binding site residues for CDK4 was identified from literature sources. Based on this information, a grid generation was performed around the ATP binding pocket of CDK4, encompassing the following residues: ILE12, VAL20, ALA33, VAL77, PHE93, GLU94, HIS95, VAL96, GLN98, ASP99, THR102, GLU144, LEU147, ALA157, and ASP158 [19].

### 2.3 Ligands preparation

12,432 approved and investigational drugs were obtained from ChEMBL library (9923 drugs) (https://www.ebi.ac.uk/chembl/) and Drug bank library (2509 drugs) (https://go.drugbank.com/). The LigPrep module of Schrodinger software was used to prepare, neutralize, desalt, and adjust tautomers of the obtained compounds and the OPLS4 force field was used to minimise their energy [18].

### 2.4 Molecular docking

The docking studies were conducted using the Glide module of Schrödinger software. Initially, Glide High Throughput Virtual Screening (HTVS) was employed to search and score all compounds obtained after the preparation of approved drugs against the receptor grid of CDK6. Subsequently, the top compounds were further subjected to docking using the Glide Extra Precision (XP) mode. The highest-ranked output from the XP docking was then docked against the receptor grid of the aromatase enzyme (PDB ID: 3S79) using the XP docking mode. Furthermore, the top-ranked output from the previous step was docked against CDK4 (PDB ID: 2W96).

To facilitate a comparison with the docking results obtained for these approved drugs, the same screening process was applied to an approved CDK4/6 inhibitor (abemaciclib) and an aromatase inhibitor (letrozole).

### 2.5 Molecular Mechanics energies combined with Generalized Born and Surface Area (MM-GBSA)

The Prime MM-GBSA module in the Schrodinger software was employed to determine the free energy associated with the binding of ligands to a protein. Which is determined by the following equation:

$$\Delta \text{E}={\text{E}}_{\text{c}}-{\text{E}}_{\text{R}}-{\text{E}}_{\text{L}}$$

where ΔE is the free binding energy, E_c_ is the target/ligand complex energy, E_R_ is the receptor energy and E_L_ is the ligand energy. The calculations were performed using the OPLS4 force field and the VSGB solvation model [20]. MM-GBSA calculations were carried out on the highest-scoring drugs obtained from docking studies, which exhibited superior scores with three specific protein targets. To compare the results of the MM-GBSA analysis, a similar screening approach was utilized for approved inhibitors targeting CDK4/6 (abemaciclib) and aromatase (letrozole).

### 2.6 Molecular dynamics (MD) simulation and post-MD MM-GBSA calculations

Desmond software was utilized to perform MD simulations for the top selected drug interacting with CDK6, based on their MM-GBSA scores, with three protein targets. The biological system was solvated using the TIP3P water model in an orthorhombic box with dimensions of 10 × 10 × 10 Å. Sodium (Na+) and chloride (Cl-) ions were added to neutralize charges. The OPLS4 force field was employed to minimize the system’s energy until reaching a threshold of 25 kcal/mol/Å. The MD simulations were conducted in the NPT ensemble class, maintaining a constant temperature of 300 K and pressure of 1 atm throughout the process. Each system was simulated for 100 ns, and 1000 frames were collected during the simulations to assess system stability. To evaluate the behaviour of each system, the MD trajectories were analysed using Desmond’s simulation interaction diagram. This analysis facilitated the generation of the root mean square deviation (RMSD) and the root mean square fluctuation (RMSF) values for both the ligand and protein, as well as their respective contacts. Moreover, the Prime module of Schrödinger was utilized to calculate the binding free energies of the MD conformations of the selected compound using the MM-GBSA continuum solvent model which incorporates the OPLS force field, VSGB solvent model, and rotamer search algorithms. The MD conformations were extracted from the MD trajectory every 200 ns with a total of 6 frames and the average was then calculated.

## 3. Results

**Fig 1:**
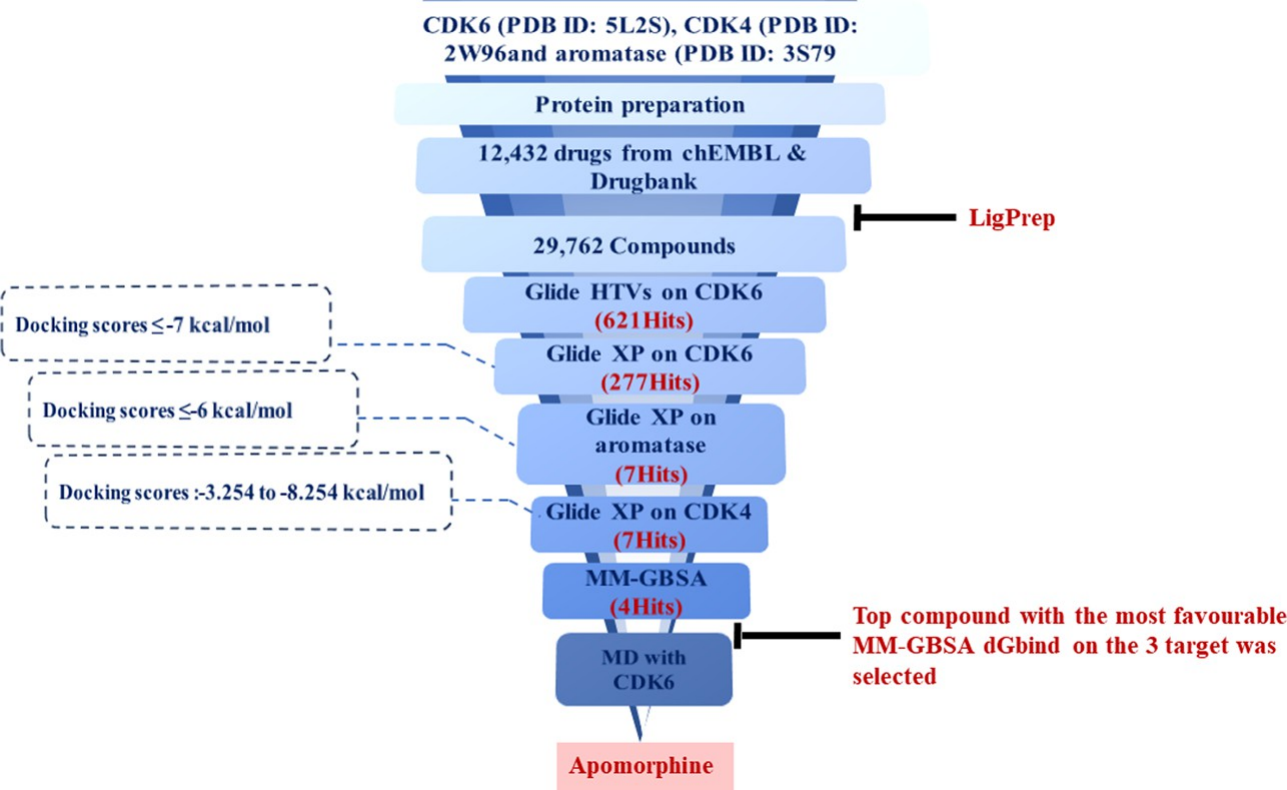
The workflow of the study. 12,432 approved were docked into the active site of CDK6 using HTVS and XP docking modes of Glide resulting in 277 compounds with docking scores ≤7 kcal/mol. These compounds were then docked against aromatase and CDK4 enzymes using XP mode and their MM-GBSA was calculated. Four drugs showed good binding affinity to all three protein targets and favourable binding free energy with CDK6. Apomorphine displayed the most favorable binding free energy with all three protein targets and the stability of its interaction with CDK6 was proven *via* molecular dynamics simulation.

The workflow of this study is summarized in Fig 1.

### 3.1 Molecular docking and MM-GBSA calculations

A total of 12,432 approved and investigational drugs from ChEMBL and Drug Bank were prepared, resulting in 29,762 compounds, including tautomers, conformers, and stereoisomers. Molecular docking was conducted on all these compounds to assess their affinity towards CDK6 (PDB ID: 5L2S) using the Glide High Throughput Virtual Screening (HTVS). Among them, 621 compounds bound to CDK6 with a docking energy of -7 kcal/mol or lower. Subsequently, these compounds were further docked against CDK6 using XP mode, resulting in 277 compounds with docking energy of -7 kcal/mol or higher. These compounds exhibited interactions with the amino acid residues responsible for CDK6 inhibitory activity (Table 1). Furthermore, these shortlisted compounds were also docked against the aromatase enzyme (PDB ID: 3S79) using the XP mode. Among them, seven drugs showed a docking score of -6.001 kcal/mol or higher (Table 1). The short-listed drugs, namely esculin, ellagic acid, trifluridine, brivudine, carazolol, dantron, and apomorphine, were then docked against CDK4 (PDB ID: 2W96) using XP mode. The results indicated that all seven drugs bound to CDK4 with docking scores ranging from -3.254 to -8.254 kcal/mol.

**Table 1:** Docking score and MM-GBSA results for the top selected drugs and references on the three target proteins.

Drug	CDK6 Docking Score kcal/mol	CDK6 MMGBA kcal/mol	CDK4 Docking Score kcal/mol	CDK4 MMGBSA kcal/mol	Aromatase Docking Score kcal/mol	Aromatase MMGBSA kcal/mol
Abemaciclib	-12,121	-68,65	-3.480	-29.60	-	-
Letrozole	-	-	-	-	-3.264	-5.06
Esculin	-12.281	-49.79	-8.254	-37.95	-9.903	-0.40
Ellagic acid	-10.825	-51.92	-6.949	-42.97	-10.403	-46.53
Trifluridine	-8.599	-29.32	-7.980	-31.75	-8.045	-18.85
Brivudine	-8.560	-38.24	-6.641	-40.88	-7.879	-19.41
Carazolol	-7.671	-53.90	-7.935	-32.56	-10.019	-4.63
Dantron	-7.592	-50.22	-7.759	-39,23	-7.694	-16.63
Apomorphine	-7.121	-60.97	-3,254	-46,41	-6,001	-36.88

**Fig 2:**
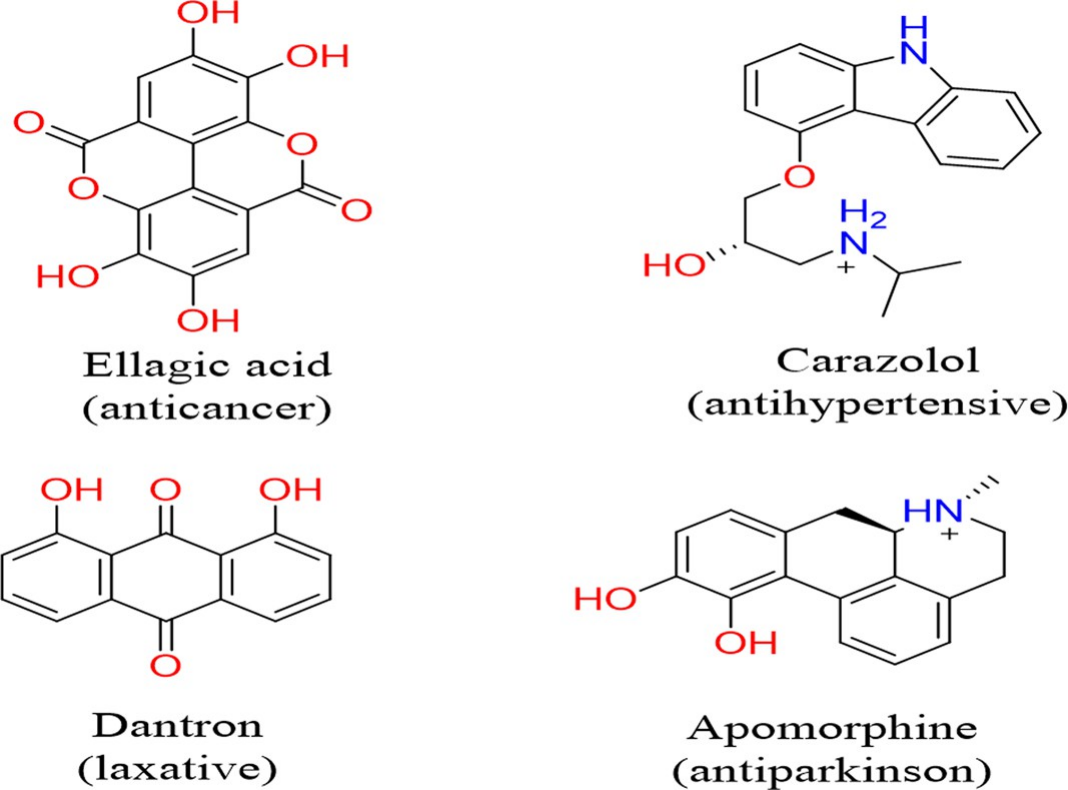
The chemical structure of the top four drugs.

The binding free energy of protein-ligand complexes for the top seven drugs was calculated using MM-GBSA based on docking scores. The obtained dGbind values ranged from -29.32 to -60.97 kcal/mol for CDK6, -31.75 to -48.71 kcal/mol for CDK4, and -0.40 to -39.06 kcal/mol for the aromatase enzyme. Among these drugs, ellagic acid, carazolol, dantron, and apomorphine showed more negative dGbind values than the others in the case of CDK6 (Table 1 and Fig 2).

The reference ligand, abemaciclib, was docked onto CDK4 and CDK6 using the G-XP mode, resulting in docking scores of -3.480 and -12.121 kcal/mol, and MM-GBSA dGbind values of -29.60 and -68.65 kcal/mol, respectively. Letrozole, a known aromatase enzyme inhibitor, displayed a docking score of -3.264 and an MM-GBSA dGbind value of -5.06 kcal/mol (Table 1).

#### 3.1.1 Analysis of intermolecular interactions

The intermolecular interactions of the four shortlisted drugs with the three targets were further analysed and compared to their reference ligands.

In CDK6, abemaciclib interacted with VAL101 through its 2-aminopyrimidine and pyrimidine nitrogen and formed two hydrogen bonds. Moreover, the benzimidazole, piperazine and pyridine nitrogen of abemaciclib formed direct and indirect hydrogen bond interactions with LYS43, ASP104 and His100.

**Fig 3:**
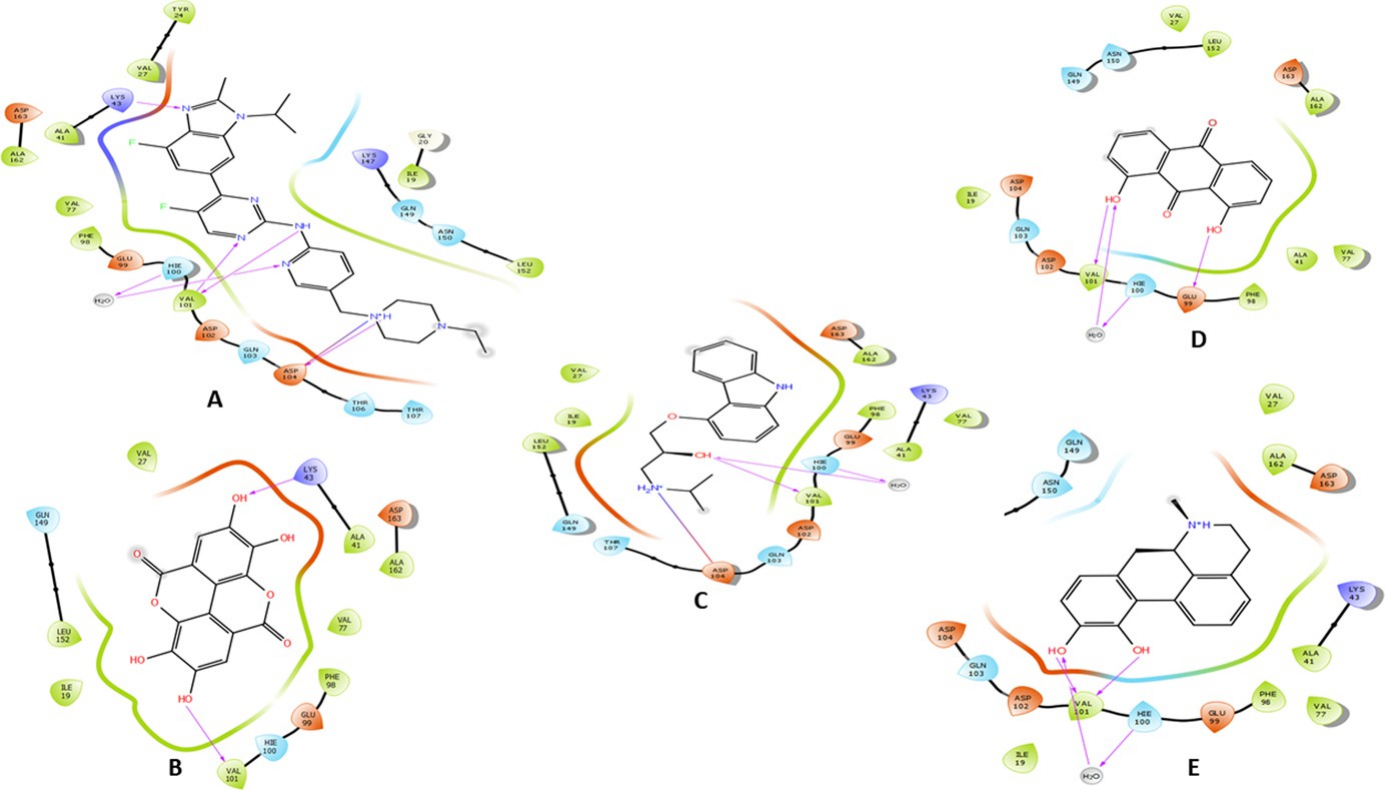
The two-dimensional (2D) interactions of (A) Abemaciclib, (B) Ellagic acid, (C) Carazolol, (D) Dantron and (E) Apomorphine with CDK6. The hydrogen bond interactions with residues are represented in a purple arrow, while the salt bridge is represented by a red-blue arrow, hydrophobic interactions happen with green residues, polar interaction with faint blue residues, positive interactions with dark blue residues and negative interaction with red residue.

**Fig 4:**
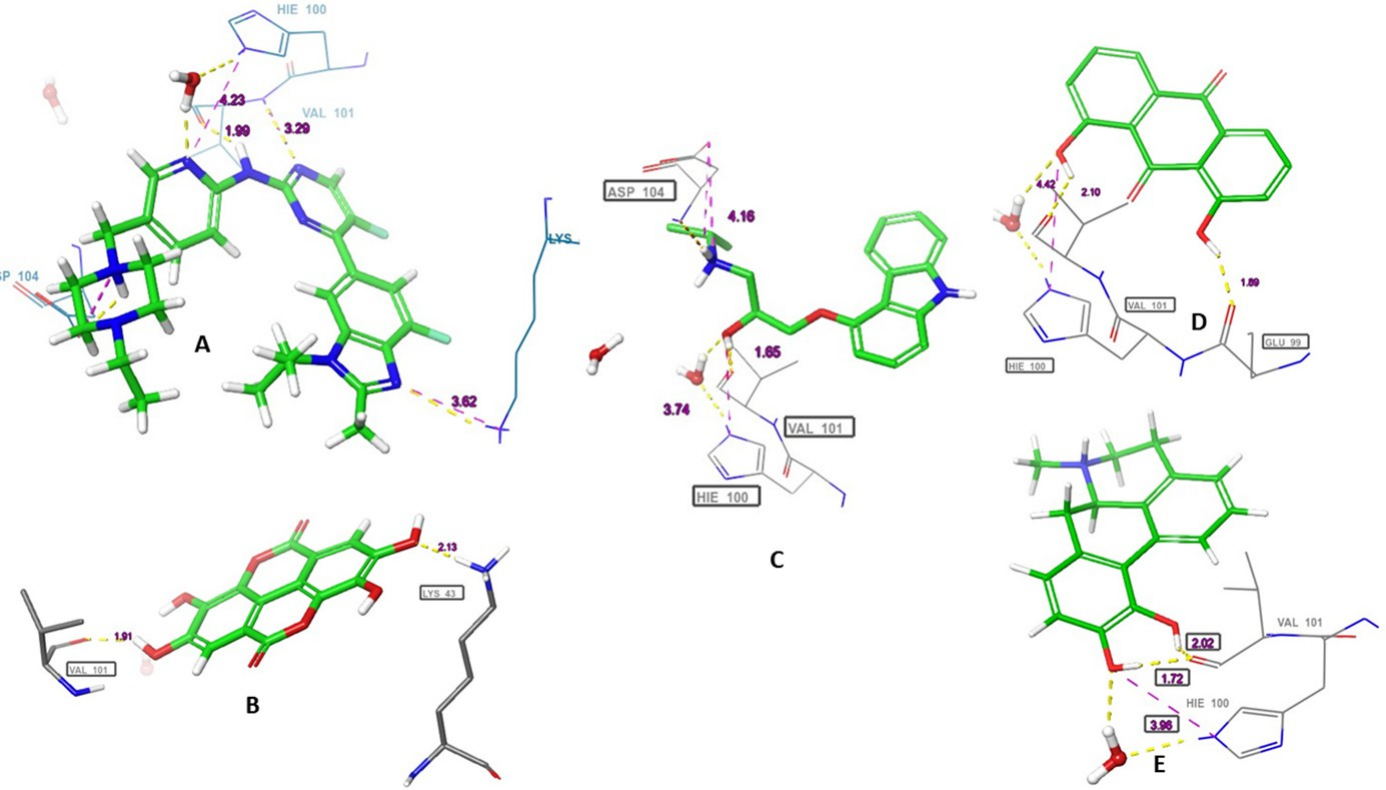
The three-dimensional (3D) interactions of (A) Abemaciclib, (B) Ellagic acid, (C) Carazolol, (D) Dantron and (E) Apomorphine with CDK6. The hydrogen bond interactions with residues are represented in purple dashes, while the salt bridge is represented in red dashes. The bond length (Å) is represented by purple numbers.

Hydrogen bonds with the carbonyl of VAL101 through the ligands phenolic hydroxyl groups were the common interaction exhibited by the four shortlisted drugs. While hydrogen bond with the NH_3_ group of LYS43 was observed with ellagic acid. Apomorphine, dantron and carazolol hydroxyl groups interacted *via* bridge hydrogen bonds with the imidazole of hinge residue HIS100. While interaction with ASP 104 was formed with the carazolol amino group. A hydrogen bond with GLU99 was established with dantron’s phenolic group. Furthermore, Van der Waals interactions engaging the ATP binding pocket key residues were observed with the reference and the short-listed ligands. (Table 2 and Figs 3 and 4).

**Table 2:** The intermolecular interactions of the shortlisted drugs and abemaciclib with CDK6.

Drug	Hydrogen bond			Salt bridge			Hydrophobic interaction	Other interaction
	Residue	Length (Å)	Angle	Residue	Length (Å)	Angle		
Abemaciclib	LYS43	2.68	154.1°	ASP104	3.01	39°	ILE19, TYR24, VAL27 ALA41, VAL77, PHE98, VAL101, LEU152, ALA162.	**Polar interaction:** HIS100, GLN103, THR106, THR107, GLU149, ASN150 **Charged positive:** LYS43, LYS143 **Charged negative:** Glu99, ASP102, ASP104, ASP163
	HIS100 (water bridge)	2.00, 1.97	90.9°					
	VAL101	1.99	145.3°					
	VAL101	2.45	140.7°					
	ASP104	2.31	124.9°					
Ellagic acid	LYS43	2.13	150.7°	-			ILE19, VAL27 ALA41, VAL77, PHE98, VAL101, LEU152, ALA162	**Polar interaction:** HIS100, GLU149 **Charged positive:** LYS43. **Charged negative:** Glu99, ASP163
	VAL101	1.91	146.3°					
Carazolol	HIS100 (water bridge)	2.00, 1.98	77.7°	ASP104	4.44	67.3°	ILE19, VAL27 ALA41, VAL77, PHE98, VAL101, LEU152, ALA162	**Polar interaction:** HIS100, GLN103, THR107, GLU149 **Charged positive:** LYS43 **Charged negative:** Glu99, ASP102, ASP104, ASP163
	VAL101	1.65	171°					
Dantron	GLU99	1.89	157.5°				ILE19, VAL27 ALA41, VAL77, PHE98, VAL101, LEU152, ALA162	**Polar interaction:** HIS100, GLN103, GLU149, ASN150 **Charged negative**: Glu99, ASP102, ASP104, ASP163
	HIS100 (water bridge)	2.00, 2.69	82.7°					
	VAL101	2.10	132.9°					
Apomorphine	HIS100 (water bridge)	2.00, 2.14	80.4°				ILE19, VAL27 ALA41, VAL77, PHE98, VAL101, LEU152, ALA16	**Polar interaction:** HIS100, GLU149, ASN150 **Charged positive:** LYS43. **Charged negative:** Glu99, ASP104, ASP163
	VAL101	2.02	140.7°					
	VAL101	1.72	167.2°					

**Fig 5:**
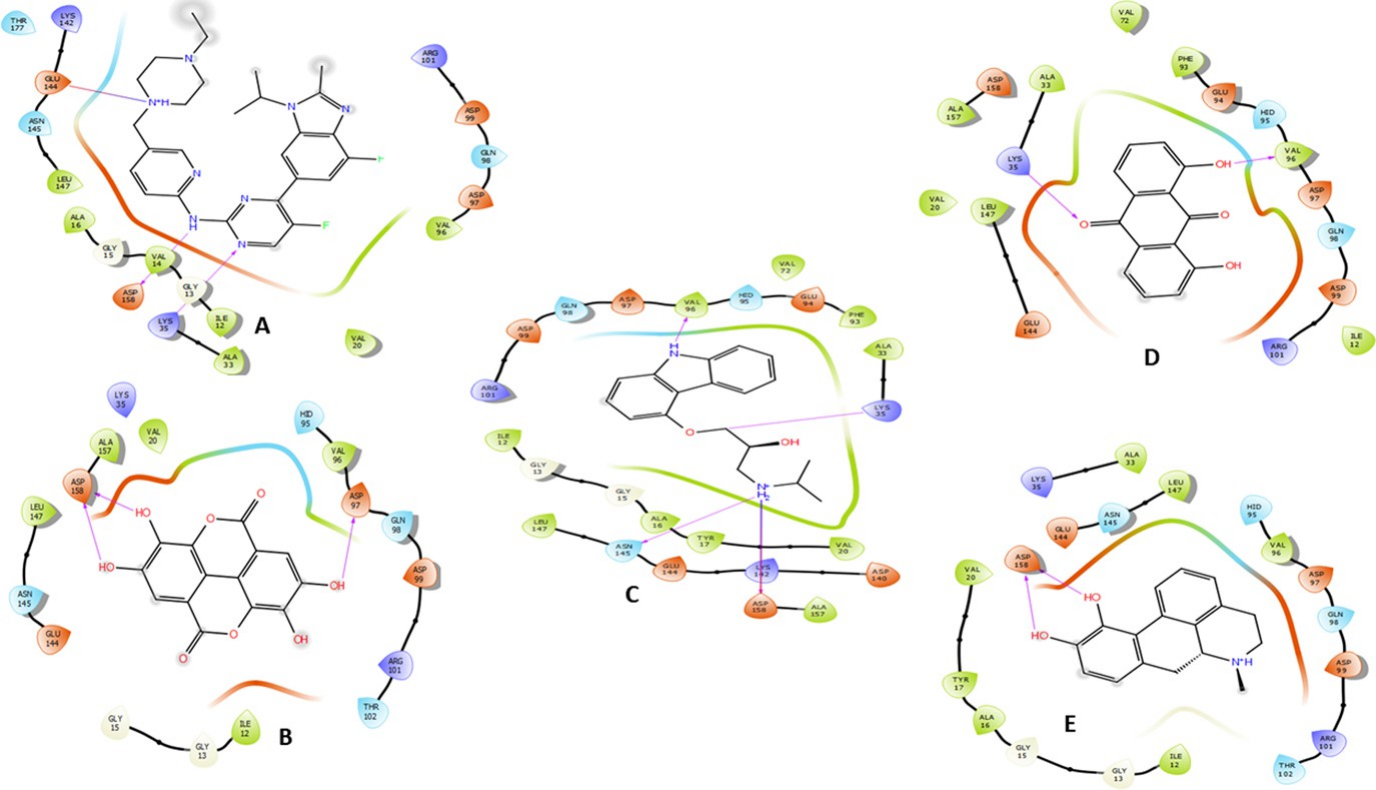
The two-dimensional (2D) interactions of (A) Abemaciclib, (B) Ellagic acid, (C) Carazolol, (D) Dantron and (E) Apomorphine with CDK4. The hydrogen bond interactions with residues are represented in a purple arrow, while the salt bridge is represented by a red-blue arrow, hydrophobic interactions happen with green residues, polar interaction with faint blue residues, positive interactions with dark blue residues and negative interaction with red residue.

**Fig 6:**
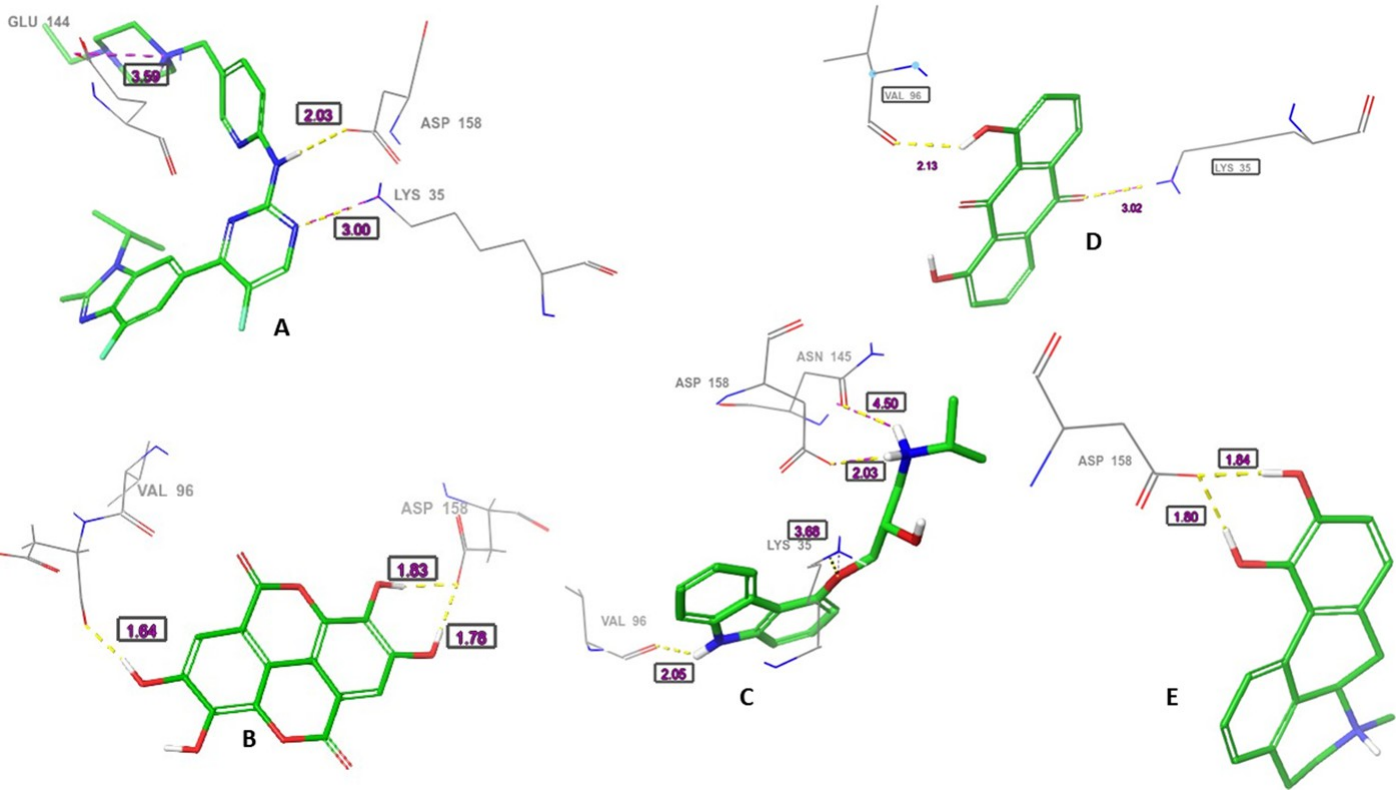
The three-dimensional (3D) interactions of (A) Abemaciclib, (B) Ellagic acid, (C) Carazolol, (D) Dantron and (E) Apomorphine with CDK4. The hydrogen bond interactions with residues are represented in purple dashes, while the salt bridge is represented in red dashes. The bond length (Å) is represented by purple numbers and the bond angle is represented by green numbers.

In CDK4, abemaciclib, ellagic acid, carazolol and apomorphine interacted through their amino and phenolic hydroxyl groups with the carbonyl of ASP158. The pyrimidine nitrogen, ether oxygen and carbonyl oxygen enable abemaciclib, carazolol and dantron, respectively to form hydrogen bonds with the NH_3_ group of LYS35. Hydrogen bonds with the VAL96 carbonyl group were mediated by the carbazole nitrogen and the phenolic hydroxyl of carazolol and dantron, respectively. Additional hydrogen bonds were observed between ASP97 and ellagic acid; ASN 145 and carazolol. (Table 3 and Figs 5 and 6).

**Table 3:** The intermolecular interactions of the shortlisted drugs and abemaciclib with CDK4.

Drug	Hydrogen bond			Salt bridge			Hydrophobic interaction	Other interaction
	Residue	Length (Å)	Angle	Residue	Length (Å)	Angle		
Abemaciclib	LYS35	2.00	169.9	GLU144	3.59		ILE12, VAL14, ALA16, VAL20, ALA33, VAL96, LEU147	**Polar interaction:** GLN98, ASN145, THR177 **Charged positive:** LYS35, ARG101, LYS142 **Charged negative:** ASP97, ASP99, ASP158, GLU144
	ASP158	2.03	175.7					
Ellagic acid	ASP97	1.64	168.3°				ILE12, VAL20, VAL96, LEU147, ALA157	**Polar interaction:** HID95, GLN98, ASN145, THR102 **Charged positive:** LYS35, ARG101 **Charged negative:** ASP97, ASP99, GLU144, ASP158
	ASP158(2)	1.83	158.9°					
		1.78	168°					
Carazolol	GLY35	2.73	136.8°	ASP158	2.95	20.4°	ILE12, ALA16, TYR17, VAL20, ALA33, VAL72, PHE93, VAL96, LEU147, ALA157	**Polar interaction:** HID95, GLN98, ASN145 **Charged positive:** LYS35, ARG101, LYS142 **Charged negative:** GLU94, ASP97, ASP99, GLU144, ASP158
	VAL96	2.05	141.8°					
	ASN145	1.95	133.8°					
	ASP158	2.03	149.6°					
Dantron	GLY35	2.09	152.2°				ILE12, VAL20, ALA33, VAL72, PHE93, VAL96, LEU147, ALA157	**Polar interaction:** HID95, GLN98 **Charged positive:** LYS35, ARG101 **Charged negative:** GLU94, ASP97, ASP99, GLU144, ASP158
	VAL96	2.13	138.6°					
Apomorphine	ASP158	1.84	166.9°				ILE12, ALA16, TYR17, VAL20, ALA33, VAL96, LEU147,	**Polar interaction:** HID95, GLN98, THR102 ASN145 **Charged positive:** LYS35, ARG101 **Charged negative:** ASP97, ASP99, GLU144, ASP158
	ASP158	1.80	162.3°					

The carazolol’s and apomorphine’s amino groups coordinate the heme iron of the aromatase enzyme. Apomorphine formed three other hydrogen bonds with the carbonyls of LEU372, 477, NH of MET 374 and one π- π interaction with TRP224. Carazolol and dantron established π- π interaction with PHE134 and TRP224. While the phenolic hydroxyls of ellagic mediated hydrogen bonds with the backbone nitrogen and carbonyl of MET 374 and LEU477, respectively.

**Fig 7:**
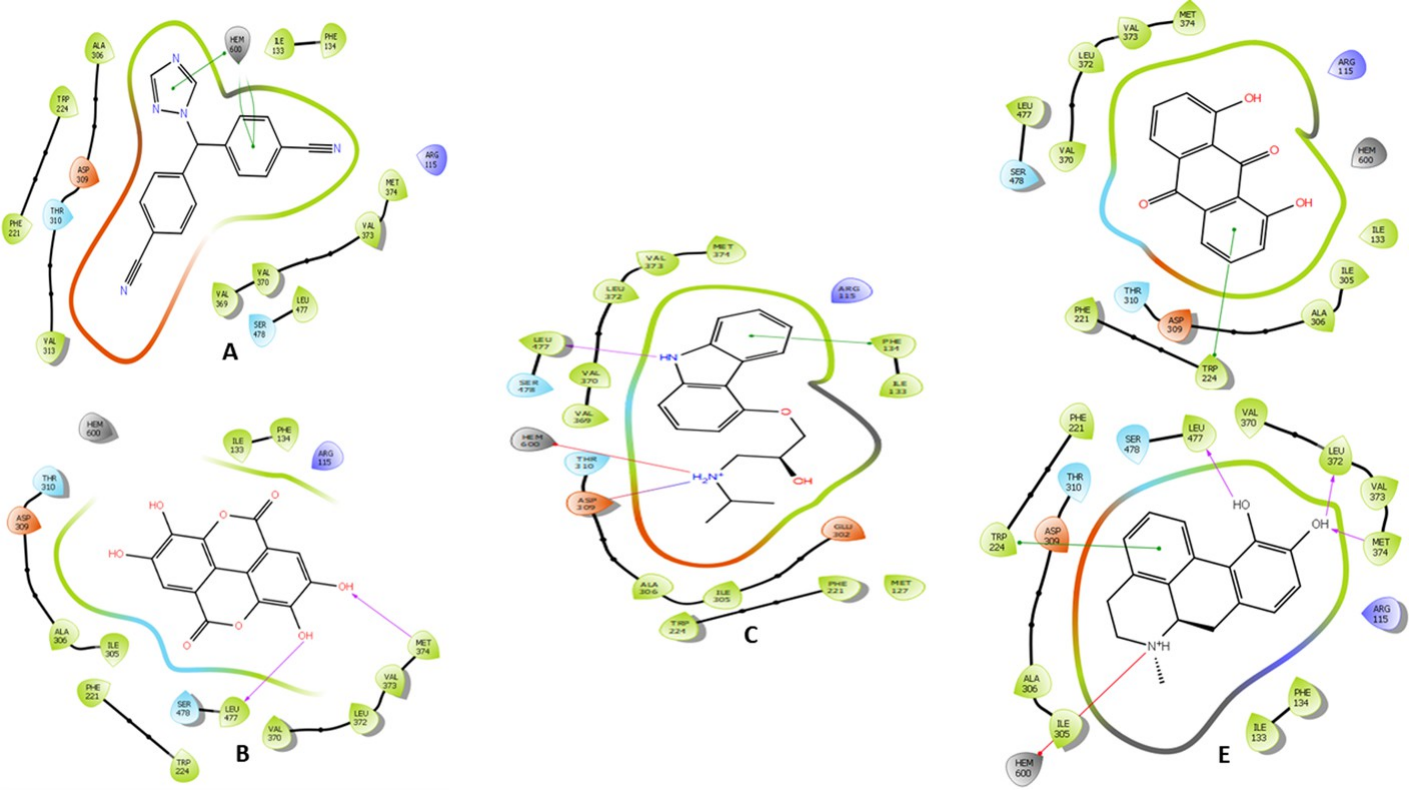
The two-dimensional (2D) interactions of (A) Letrozole, (B) Ellagic acid, (C) Carazolol, (D) Dantron and (E) Apomorphine with Aromatase enzyme. The hydrogen bond interactions with residues are represented in a purple arrow, while the salt bridge is represented by a red-blue arrow, the pi-cation interaction is represented by a red line and the pi-pi interaction is represented by a green line. Hydrophobic interactions happen with green residues, polar interaction with faint blue residues, positive interactions with dark blue residues and negative interaction with red residues.

**Fig 8:**
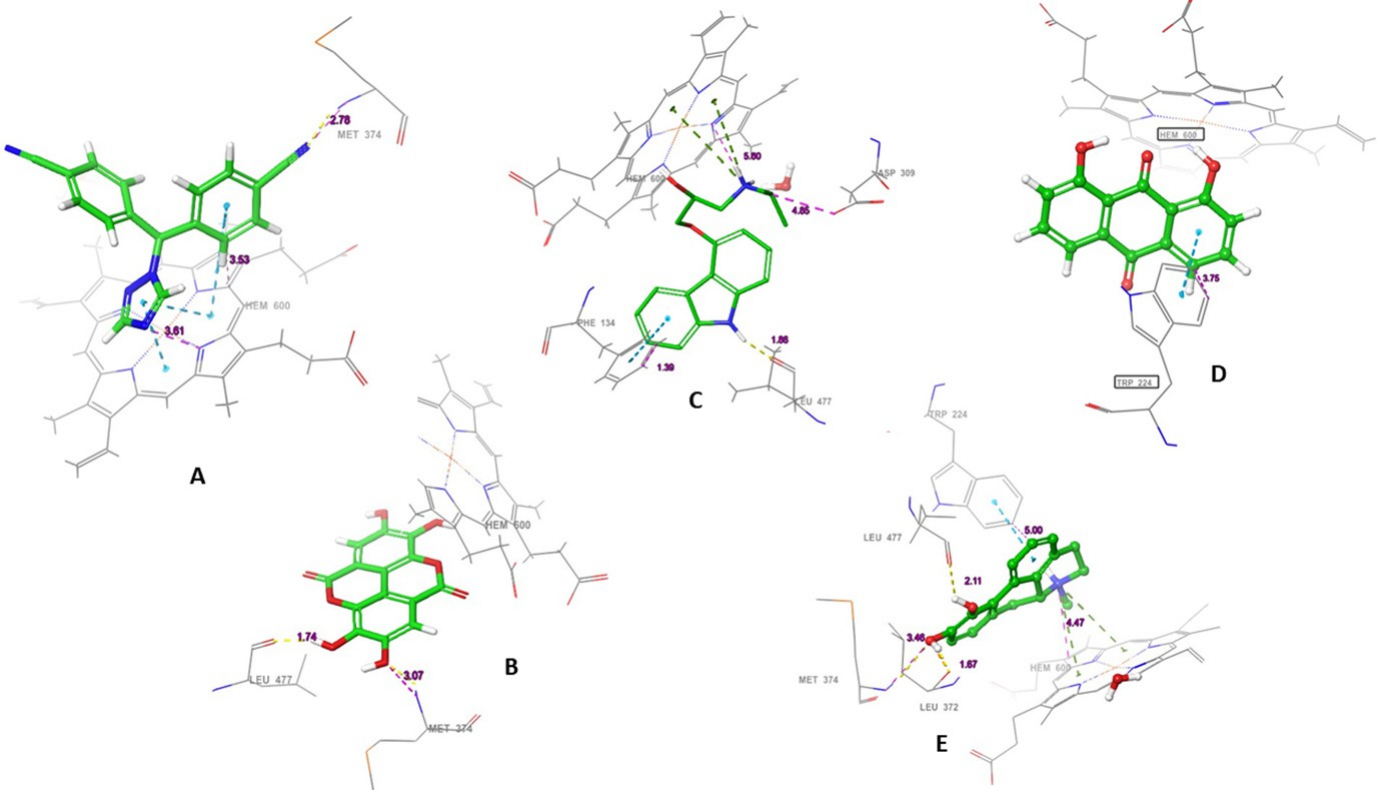
The three-dimensional (3D) interactions of (A) Letrozole, (B) Ellagic acid, (C) Carazolol, (D) Dantron and (E) Apomorphine with Aromatase enzyme. The hydrogen bond interactions with residues are represented in purple dashes, while the salt bridge is represented in red dashes. The bond length (Å) is represented by purple numbers.

On the other hand, letrozole formed three pi-pi bonds with the heme iron. The shortlisted compounds and the reference ligand formed Van der Waals interactions with the residues comprising the active cleft of aromatase; PHE221, TRP224 ALA306, ALA307, VAL370, LEU372, MET374 and LEU477. (Table 4 and Figs 7 and 8).

**Table 4:** The intermolecular interactions of the four selected drugs and letrozole with aromatase enzyme.

Drug	Hydrogen bond			Pi-Pi interaction			Hydrophobic interaction	Other interaction
	Residue	Length (Å)	Angle	Residue	Length (Å)	Angle		
Letrozole				HEM600	4.8		ILE133, PHE134, PHE221, TRP224, ALA306, ALA313, VAL369, VAL370, VAL373, MET374, LEU477	**Polar interaction:** THR310, SER478 **Charged positive:** ARG 115 **Charged negative:** ASP309
				HEM600	5.21			
				HEM600	5.13			
Ellagic acid	MET374	2.16	148.3°				ILE133, PHE134, PHE221, TRP224, ILE305, ALA306, VAL370, LEU372, VAL373, MET374, LEU477	**Polar interaction:** THR310, SER478 **Charged positive:** ARG 115 **Charged negative:** ASP309
	LEU477	1.74	153.7°					
Carazolol	LEU477	1.81	174.2°	PHE134	5.30		MET127, ILE133, PHE134, PHE221, TRP224, ILE305, ALA306, VAL369, VAL370, LEU372, VAL373, MET374, LEU477	**Polar interaction:** SER478 **Charged positive:** ARG 115 **Charged negative:** ASP309, GLU302 **Pi-cation:** HEM600 (5.47 Å) **Salt bridge:** ASP309 (4.85Å, 107.2°)
Dantron				TRP224	4.80		ILE133, PHE221, TRP224, ILE305, ALA306, VAL370, LEU372, VAL373, MET374, LEU477	**Polar interaction:** THR310, SER478 **Charged positive:** ARG 115 **Charged negative:** ASP309
Apomorphine	LEU372	16.7	132°	TRP224	5.01		ILE133, PHE134, PHE221, TRP224, ILE305, ALA306, VAL370, LEU372, VAL373, MET374, LEU477	**Polar interaction:** THR310, SER478 **Charged positive:** ARG 115 **Charged negative:** ASP309 **Pi-cation:** HEM600(4.84 Å)
	MET374	2.07	162.8°					
	LEU477	2.11	128.8°					

### 3.2 MD Simulation and post-MD MM-GBSA calculations

Apomorphine exhibited a strong binding affinity for all three protein targets and had the most favourable MM-GBSA dGbind compared to the other drugs (Table 1). Thus, MD simulations were performed to study the binding of apomorphine with CDK6, using the Desmond software. The simulations were conducted for 100 ns, and MD trajectories were saved every one hundred ps, resulting in a total of 1000 frames. These frames were analyzed using Desmond’s simulation interaction diagram.

**Fig 9:**
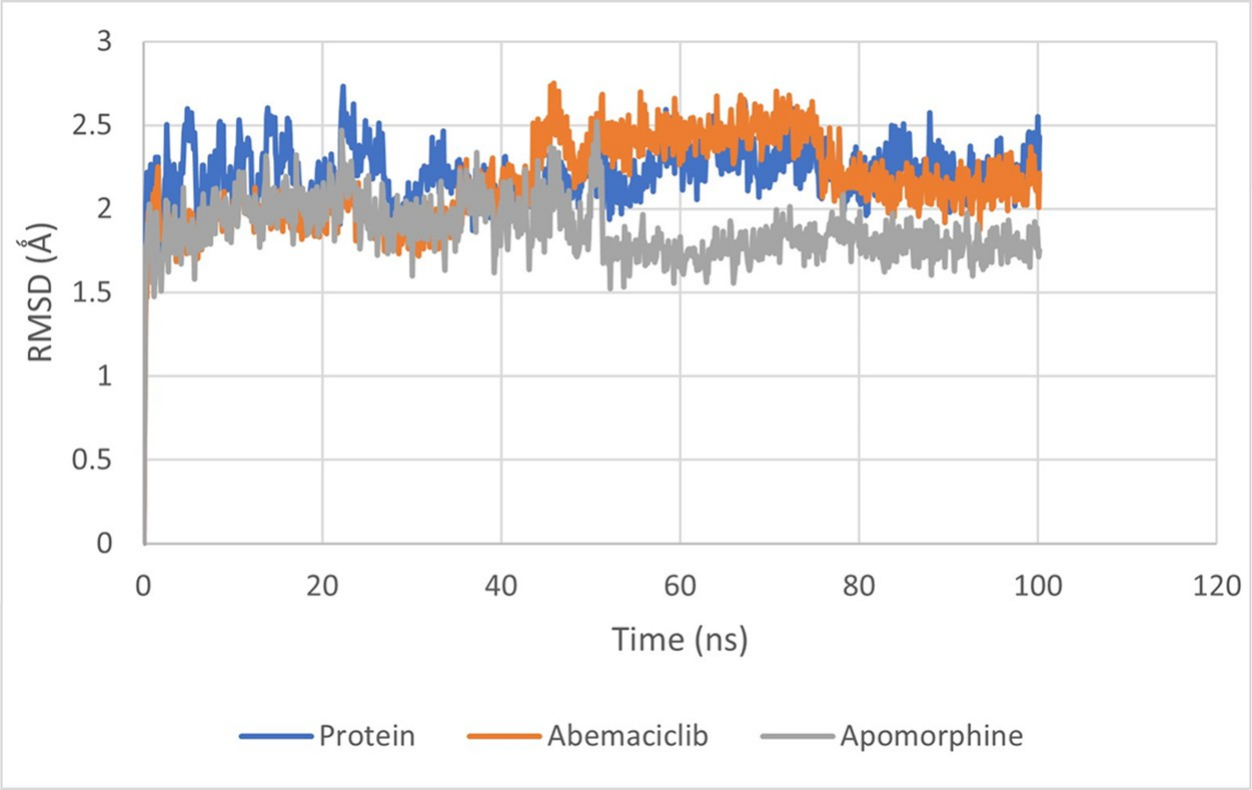
The root-mean-square deviation of apomorphine and abemaciclib complexed with CDK6 protein.

To assess the structural stability of the complexes, the root mean square deviation (RMSD) of the Cα atoms was calculated. In globular proteins, changes in the order of 1 to 3 Å are considered acceptable. The RMSD plot for the CDK6-apomorphine complex indicated that it reached equilibrium with an RMSD value of 2.1 Å, within the first 5 ns of the simulation. Subsequently, a minor drift to 1.8 Å was observed after 50 ns until the end of the simulation (Fig 9).

While Abemaciclib displayed an average RMSD of 1.8 Å. Minor fluctuations were observed around the duration of 45-80ns of the simulation (Fig 9).

**Fig 10:**
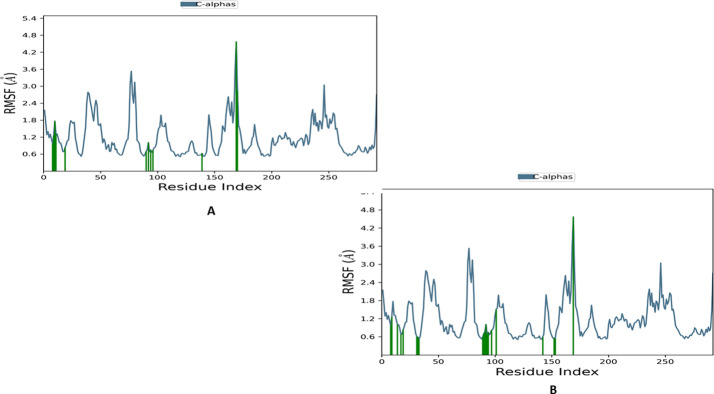
The root means square fluctuation (RMSF) plot of CDK6 complexed (A) Apomorphine and (B) Abemaciclib. Green-coloured vertical bars are indicated CDK6 residues that interact with apomorphine.

Fig 10 depicts the protein root mean square fluctuation (RMSF) data for the two complexes, showing that most residues exhibited fluctuations between 0.6 and 3 Å. However, a few residues displayed higher values exceeding 3 Å, indicating extra-conformational flexibility in those regions.

**Fig 11:**
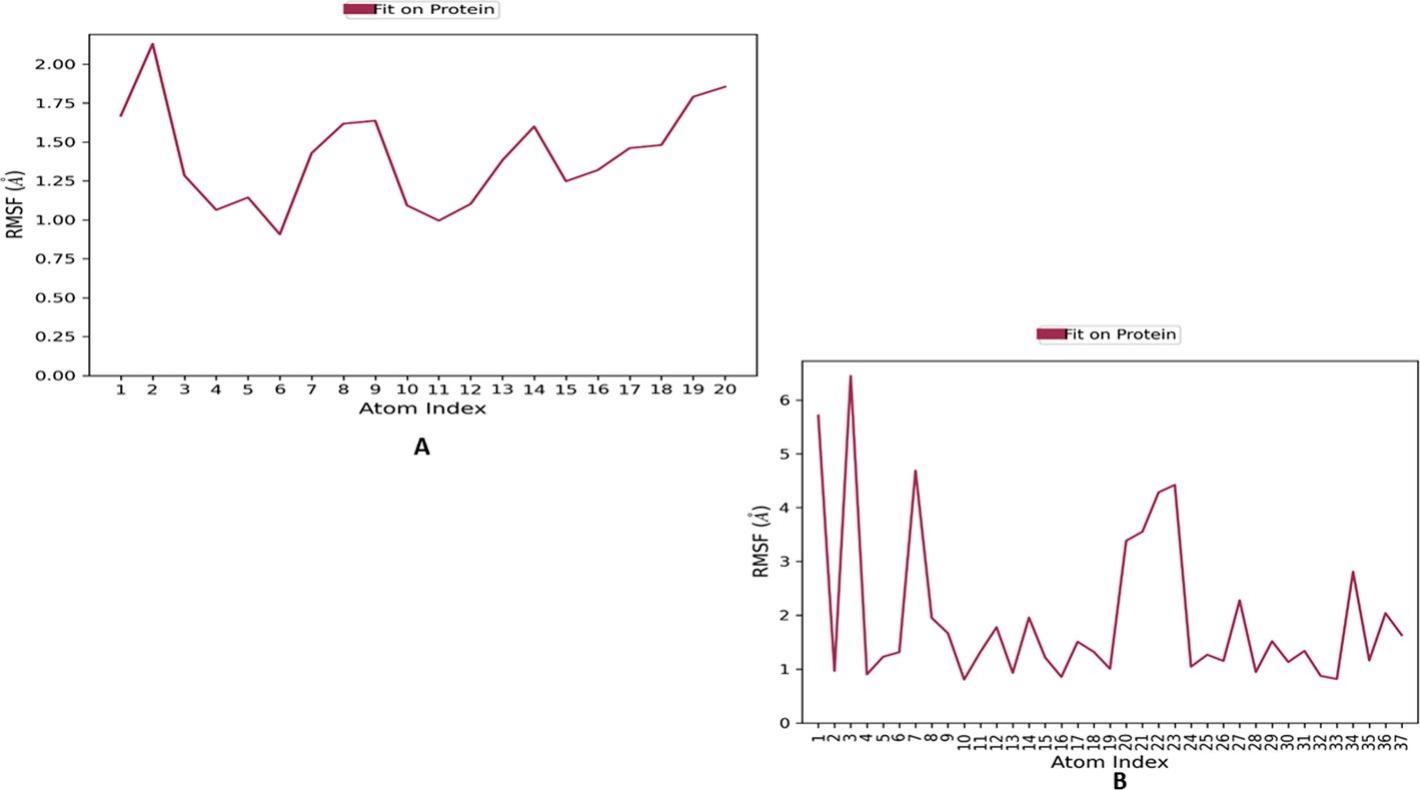
The root means square fluctuation (RMSF) plot for atoms of (A) apomorphine and (B) Abemaciclib.

The ligand RMSF was used to analyze changes in the positions of the ligand atoms. The RMSF values for apomorphine atoms were ≤1.6 Å, while for abemaciclib atoms, most RMSF values were ≤4 Å, except for atoms 1 and 3, which exhibited higher fluctuations (Fig 11).

**Fig 12:**
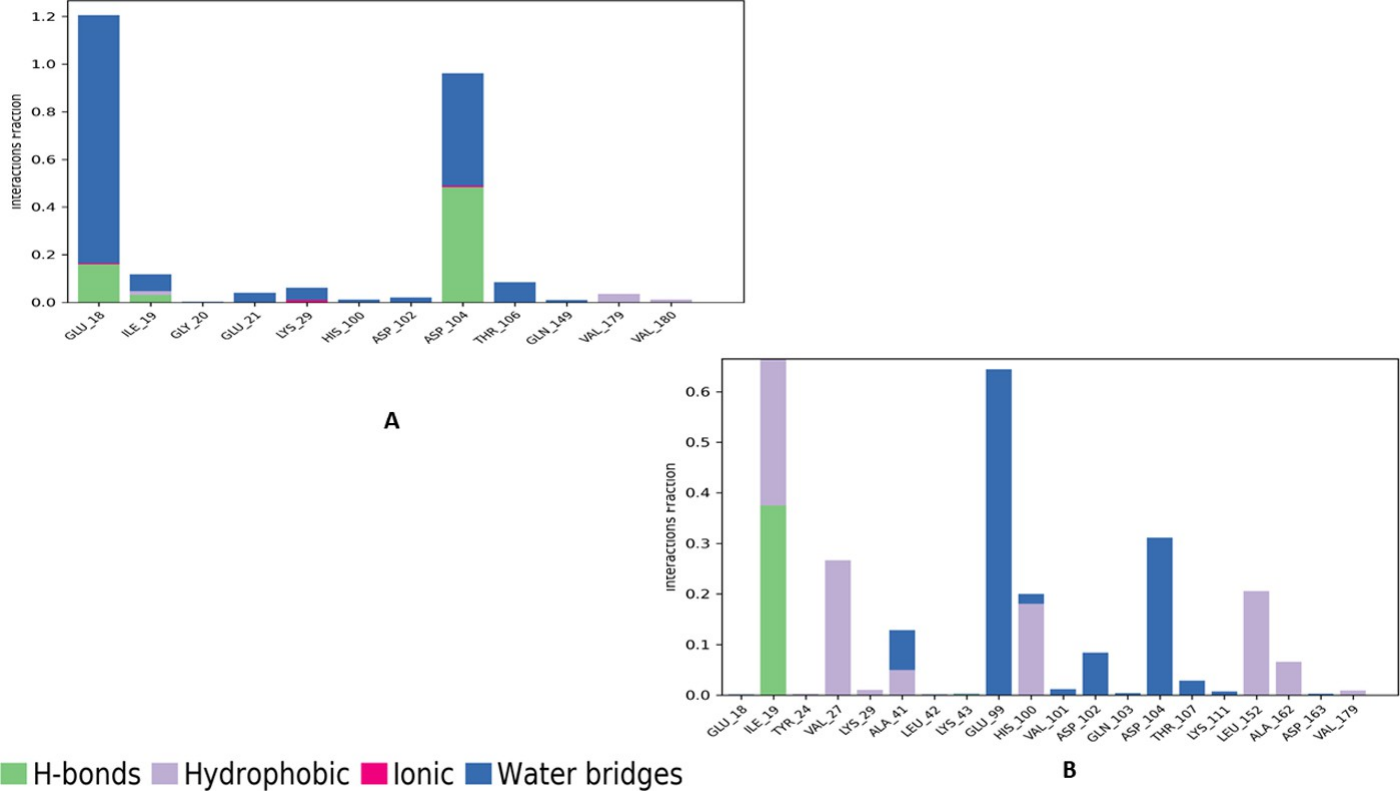
The protein-ligand contact histogram of (A) Apomorphine and (B) Abemaciclib complexed with CDK6 protein.

To identify the interactions responsible for maintaining the stability of the complexes, the interaction profile of the ligand with the protein was examined. In the CDK6-apomorphine complex, apomorphine formed direct and indirect hydrogen bonds with ASP104 (48% and 47%, respectively). Additionally, a bridged hydrogen bond was observed with GLU18 (100%) (Fig 7). For the abemaciclib complex, the ligand was bound through a direct hydrogen bond with ILE19 (37%) and bridged hydrogen bonds with GLU99 (64%) and ASP104 (30%) (Fig 12).

**Fig 13:**
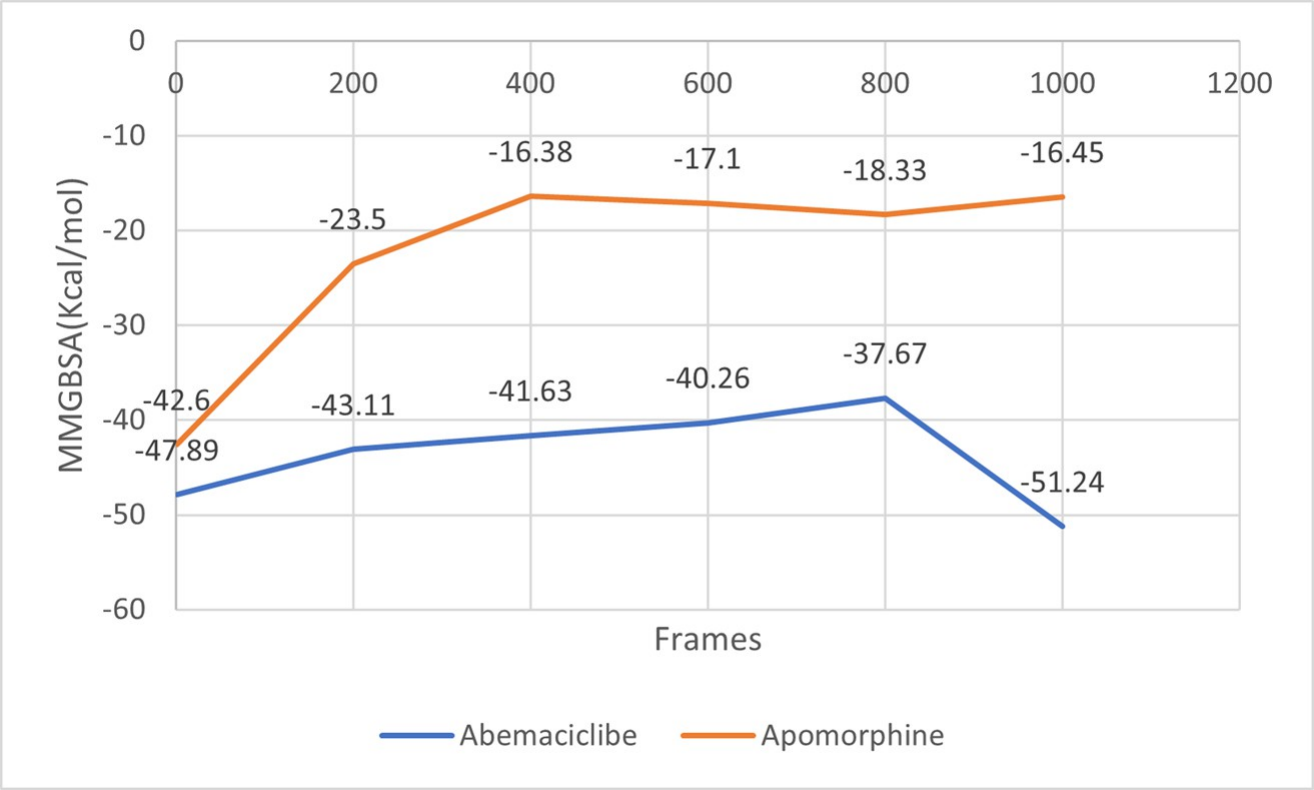
The post-molecular dynamics MM-GBSA calculations of apomorphine and abemaciclib complexed with CDK6 protein.

Post-MD MM-GBSA calculations on apomorphine and abemaciclib complexes exhibited average values of—22.39, and—43.63 kcal/mol, respectively (Fig 13).

## 4. Discussion

Inhibition of estrogen biosynthesis and uncontrolled cell proliferation by combing CDK4/6 and aromatase inhibitors is the mainstay of treatment therapeutic strategy for estrogen receptor-positive breast cancer [21, 22].

Drug repurposing is a useful approach for the identification of new anticancer agents [23]. It has advantages over traditional drug discovery because it reduces the duration and cost of the drug discovery process with highly efficient and minimal risk of failure [14]. Structural-based virtual screening is one of the target-based drug repositioning methods [13].

In this study, *in silico* techniques including molecular docking, MM-GBSA, and molecular dynamics were employed to identify compounds with multitarget inhibitory potentiality against CDK4/6 and aromatase for the treatment of estrogen receptor-positive breast cancer.

The results of molecular docking revealed that seven drugs, namely esculin, ellagic acid, trifluridine, brivudine, carazolol, dantron, and apomorphine, exhibited strong binding affinity against the three proteins. Furthermore, the binding free energy analysis showed that four drugs, specifically ellagic acid, carazolol, dantron, and apomorphine, demonstrated favourable MM-GBSA dGbind values with CDK6: -51.92, -53.90, -50.22, and -60.97 kcal/mol, respectively.

All CDK4/6 inhibitors bind with the ATP binding pocket of CDK4/6 and prevent the binding of ATP and the subsequent phosphorylation and activation of the protein [17]. The binding of these inhibitors is mediated by hydrogen bonds and several hydrophobic contacts, altogether mediating the very tight binding [17].

The hydrophobic nature of the polycyclic ellagic acid, carazolol, dantron, and apomorphine allowed them to engage VAL20, VAL96, and LEU147 and VAL27, VAL77, VAL101, LEU152, ALA162 of CDK4 and 6, respectively in hydrophobic interactions. These residues were found to be the key residues that interacted with the most CDK4/6 ATP-competitive inhibitors [17].

Moreover, the presence of hydrogen bond donors; phenolic hydroxy / 2-aminopyrimidine in the shortlisted drug and abemaciclib established hydrogen bonds with VAL101. VAL101 is critical for the recognition of inhibitors in the CDK6 binding site [24]

Interaction with the non-conserved HIS100 is a determinant of selectivity over other kinases [25]). Water-mediated hydrogen bond with HIS 100 was formed by the 3-N pyridine of abemaciclib and the hydroxyl groups of apomorphine, carazolol and dantron. ASP104 is a key residue that interacts with the most ATP-competitive inhibitors of CDK6 [17]. The charged functional group (amino) allowed carazolol and abemaciclib to interact with ASP104. Even though apomorphine contains charged amino groups; it was observed that the pose obtained after molecular docking lacked the salt bridge interaction mediated by the ASP104 carbonyl group. However direct and indirect hydrogen bonds with ASP104 were observed during the MD.

In aromatase, the polycyclic nucleus allowed the four drugs to occupy the active cleft and display hydrophobic interaction with its key residues. Moreover, the charged amino groups of carazolol and apomorphine coordinate the heme iron of the aromatase enzyme. While hydrogen bond donors (OH and NH) established hydrogen bonds between LEU372, 477, MET 374 and carazolol, apomorphine and ellagic acid.

Interestingly, these interactions with aromatase aligned with those reported in the modelling study that concluded that the N-4 atom of the triazole group coordinates the heme iron of the aromatase enzyme and LEU372, 477, MET 374 residues are essential for the binding of triazole inhibitors [26].

The reference abemaciclib and apomorphine were carried forward for MD simulation to get insight into their binding stability with CDK6. Overall, during simulation, interaction with one or more of the key residues in the binding pocket was observed and the obtained results from RMSD and RMSF revealed that these interactions have maintained the stability of the ligands in the binding pocket. However, the post-MD MM-GBSA of apomorphine showed an average value of -22.39 kcal/mol compared to -60.97 kcal/mol post-docking MM-GBSA. The post-docking MM-GBSA represents a single state of the protein-ligand interaction. This pose enabled the compound to form direct and indirect hydrogen bonds with HIS100 and VAL101 *via its* phenolic hydroxyl group. While the post-MD MM-GBSA depicts different protein-ligand conformations, these conformations lacked the interactions observed upon docking and displayed new direct and indirect hydrogen bonds with GLU18 and ASP104 which may result in the observed variability between the post-docking and post-MD MMGBSA energies.

Apomorphine is a derivative of morphine that acts as an agonist for dopamine 1 and 2 receptors. It is commonly used for the treatment of Parkinson’s and Alzheimer’s diseases [27]. Additionally, apomorphine has shown potential anticancer activity, particularly in suppressing the metastasis of brain and breast cancer through the inhibition of the ERK1/2 signaling pathway [28]. Furthermore, apomorphine has been found to have therapeutic effects against human epithelial ovarian cancer by reducing cellular viability and proliferation [29].

## 5. Conclusion

The simultaneous inhibition of CDK4/6 and aromatase has established a suitable strategy for treating ER+ breast cancer. In this study, computational techniques were employed to identify potential multitarget CDK4/6 and aromatase inhibitors. Molecular docking of drugs from the ChEMBL and Drugbank libraries was conducted, resulting in the selection of seven drugs based on their binding affinity and molecular interaction analysis. Further analysis using MM-GBSA calculations was performed on the shortlisted drugs, revealing those four drugs—ellagic acid, carazolol, dantron, and apomorphine exhibited favourable MM-GBSA dGbind values with CDK6. Notably, apomorphine displayed favourable MM-GBSA dGbind values not only with CDK6 but also with the three protein targets under investigation. To assess the binding stability of apomorphine with CDK6, MD simulations were conducted for a duration of 100 ns. The results of the simulations indicated that apomorphine exhibited good binding stability throughout the simulation period. However, variability between the post-docking and post-MD MMGBSA energies was observed, which might be attributed to the different apomorphine conformations during MD. These findings highlight the potential of apomorphine as an agent targeting CDK4/6 and aromatase and provide structural insight for further optimization.
